# Overlooked Bird Extinctions in Semideciduous Atlantic Forests

**DOI:** 10.1002/ece3.70388

**Published:** 2024-10-16

**Authors:** Vagner Cavarzere, Luís Fábio Silveira

**Affiliations:** ^1^ Departamento de Biodiversidade e Bioestatística, Instituto de Biociências Universidade Estadual Paulista Botucatu SP Brazil; ^2^ Seção de Aves Museu de Zoologia da Universidade de São Paulo São Paulo SP Brazil

**Keywords:** defaunation, forest clear‐cutting, local avian extirpations, nested endemic species composition, ornithological records, unnoticed distribution patterns

## Abstract

The semideciduous forest is a prominent yet highly degraded phytophysiognomy within the Atlantic Forest. Historically, bird species inhabiting these forests occurred throughout central and western São Paulo state, south‐eastern Brazil, until the mid‐20th century. Many of these species have not been observed again or are nearing extinction within the state's inland forest fragments. This study reviews and compares historical and recent ornithological records, using museum specimens, literature, citizen science and recent field surveys to understand these species' current lack of records. In the early 20th century, extensive deforestation occurred statewide, resulting in the current forest fragments, which are currently in various stages of regeneration, conservation and isolation. Many of these fragments lack the specific habitats some species require, or they have not been recolonised due to insufficient connectivity with other forest fragments where these species still exist (particularly in the eastern Atlantic Forest). The non‐detection of forest species in semideciduous forest fragments strongly suggests an unprecedented and largely unnoticed extinction scale. This pattern of defaunation, as predicted 30 years ago, may be prevalent across numerous Semideciduous Atlantic Forests in Brazil.

## Graphical abstract

Due to near total deforestation in much of the Brazilian state of São Paulo in the early 20th century, essentially all non‐coastal and not montane forest fragments today are regenerated. Before deforestation, most Atlantic Forest endemic bird species were found throughout the state, as demonstrated by museum specimens and the previous literature. Currently, endemics are not reported from western fragments, as illustrated by the recent literature (post‐1990s), citizen science data and our recent field inventories.

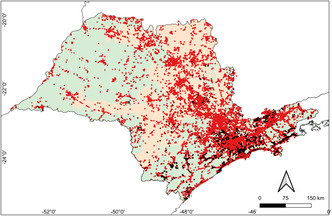

## 1 Introduction

The Atlantic Forest (AF), a biodiversity hotspot (Myers et al. 2000), extends across parts of Argentina and Paraguay, but is located mostly (83%) within Brazil. These forests are composed of several vegetation types; three forests, however, predominate: the Atlantic dense evergreen tropical forest, the *Araucaria* mixed forest and the semideciduous forest. The Atlantic dense evergreen tropical forests stretch along the eastern Brazilian coasts and mountains, occurring westward in inland Brazil. They reach the *Araucaria* mixed forests in the South and the seasonal semideciduous forests in the Southwest (Carlucci, Marcilio‐Silva, and Torezan 2021; Vancine et al. 2024). Semideciduous forests represent the largest percentage (49%) of the AF vegetation but are the most severely fragmented and least protected of all AF forest types (Vancine et al. 2024).

Deforestation in the interior (meaning inland) of the state of São Paulo rapidly accelerated after the First World War to make way for extensive coffee, followed by sugarcane and orange plantations. At that time, forests were mostly clear‐cut, with only a few large blocks of forest that were restricted to the north‐western corner by the 1960s, when only 13% of the forest remained. Currently, the Morro do Diabo State Park (> 36,000 ha) is the only sizeable semideciduous forest remnant in the state (Rezende et al. 2018). Only in the early 1970s did the Brazilian government begin to establish Forest Codes and the first public reserves (Victor et al. 2005). The distribution of protected areas in the state of São Paulo is biased towards the eastern Atlantic coast. Setting up protected areas was not random but a consequence of land use described above, which forced many conservation units to be delimited within the few remaining fragments at the time. Also, locations of larger protected areas often resulted from topography, where the mountains of the east (Serra do Mar Mountain Range) impeded other for‐profit land uses, and seldom to the west of the mountains, where the flatter lands facilitated deforestation.

Most of the semideciduous Atlantic Forest fragments in inland São Paulo are at imminent risk of disappearing forever. These fragments have been severely mistreated: they were clear‐cut in the past, subjected to random regeneration and now exist as small, isolated patches affected by edge effects (Dias, Silveira, and Francisco 2023). Embedded in a matrix of agriculture and cattle ranching, they are highly susceptible to fires and other anthropogenic activities such as continuous poisoning or logging. The ecological patterns and processes in these areas cannot be restored, as the original composition of plants and animals is lost, and the fragments are either on the verge of collapse or have already collapsed (e.g., Navarro et al. 2021). Unfortunately, the distressing experience of surveying these fragments is, unfortunately, not unique to São Paulo. In forest fragments of other severely devastated Brazilian states, such as Alagoas, even common and resilient species, such as thrushes and tanagers, are no longer found. The reality of an ‘empty forest’, if a collection of trees can still be called a forest, is a harsh and sad reality in many parts of the Brazilian Atlantic Forest (Silveira, Olmos, and Long 2003).

Today, endemic AF bird species can be found in the coastal forests, because large forest fragments persist. Such species are also found in the high plateaus west of the Serra do Mar (east of the Cerrado), even though this region is also severely fragmented (Vancine et al. 2024). It is known, based on museum specimens and earlier studies, that some of these endemic and forest species once occurred in semideciduous forests within and west of the Cerrado, such as the Red‐breasted Toucan *Ramphastos dicolorus* (Short and Sharpe 2020). Recent citizen science data have failed to detect this large, conspicuous species that was previously reported within and west of the Cerrado (Short and Sharpe 2020). This lack of records is relatively recent, with the earliest sightings within and west of the Cerrado dated from the 1990s (Pinto 1932, 1938; Willis and Oniki 2003). Here, we compared the occurrences and non‐detections of endemic bird species in semideciduous forests west of the Cerrado in São Paulo over time. We highlight an ongoing and overlooked wave of bird extinctions in the semideciduous Atlantic Forests of São Paulo state.

## 2 Methods

### 2.1 Study Areas

**FIGURE 1:**
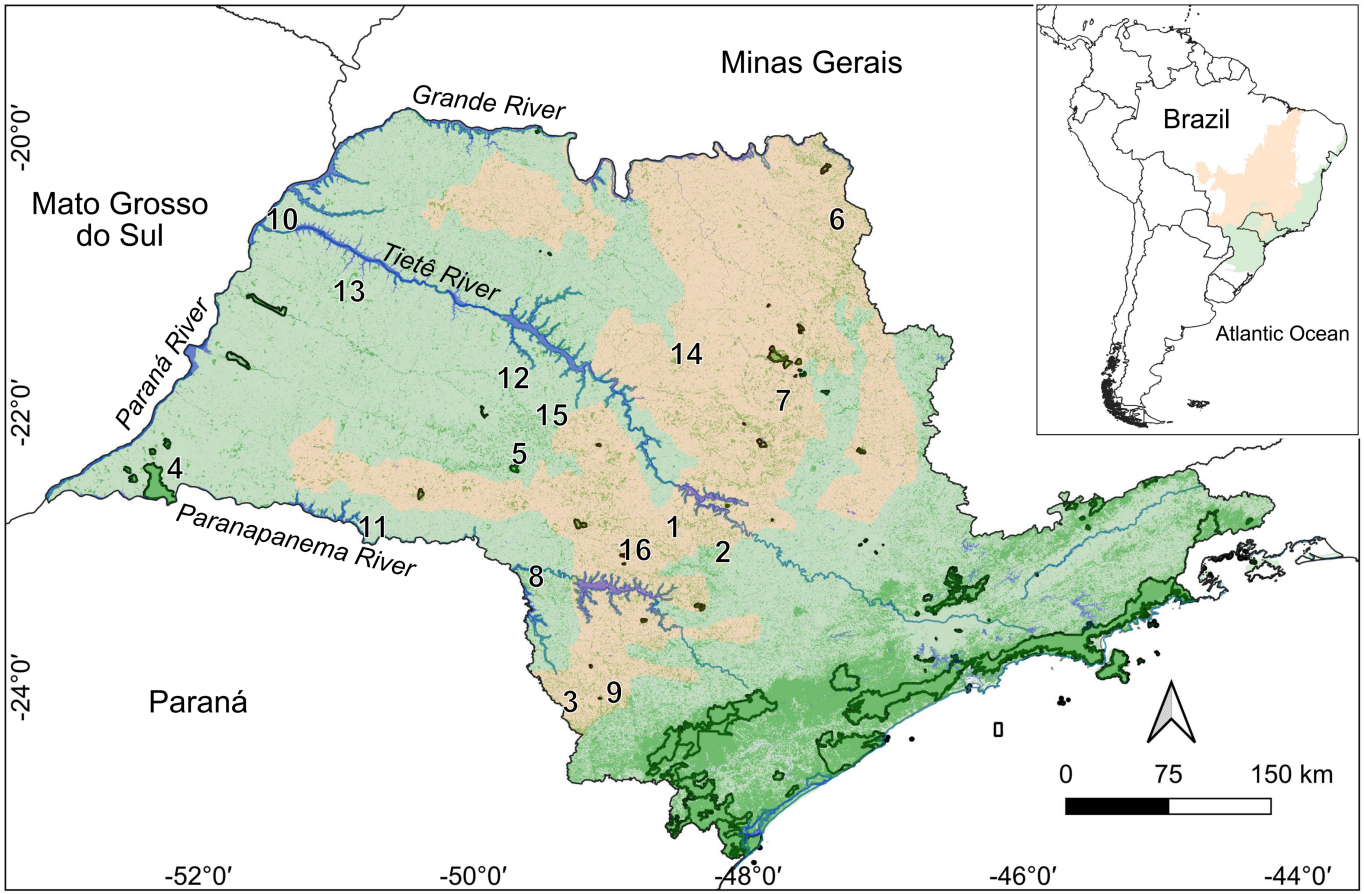
The distribution of the original Atlantic Forest (green) and Cerrado (light orange) in the state of São Paulo, south‐eastern Brazil. Delimited areas are state integrally protected areas.

São Paulo, between 19°45′ S, 53°30′ W and 25°18′ S, 44°00″ W, south‐eastern Brazil (~250,000 km^2^) has its highest elevations along the eastern coast (Serra do Mar), reaching up to 1539 m, with some high (800–1000 m) plateaus on the western side of that range. As one moves westward, altitude decreases to 300–400 m along the Paraná River (Alvares et al. 2013). Annual mean temperatures are mild (12°C–16°C) at higher elevations and hotter (20°C–26°C) in the lower, western, region. More relevant total annual rainfall (1600–2500 mm) is also along the eastern coast, while a more seasonal climate is accompanied by less rainfall (1000–1600 mm) in the lower, midwestern plateaus (Alvares et al. 2013). There are two kinds of AF in São Paulo: tropical rainforests in the mountainous coastline and semideciduous forests in the flatter and drier interior, where about half the tree species drop their leaves during the driest months of the year (Alvares et al. 2013; IBGE 2012). The Cerrado extends into the state from the north, moving southward, and divides the state into two distinct sections (Figure 1).

### 2.2 Sites Selection

We divided the state of São Paulo into 33 quadrants (2° each), in which at least one forest fragment was the objective of bird censusing. We did not include eastern quadrants with continuous forests, given they are known to have all endemic bird species. Initially, we attempted to use random fragment sampling per quadrant, but that was not possible due to terrain inaccessibility or lack of trails inside the forests. We then focused on the largest and most easily accessible fragments in central quadrants, with a total of 38 municipalities (45 forest fragments) visited in São Paulo (Table 1). We intended to visit all sites three times consecutively during the austra reproductive season between 2015 and 2016. However, due to uncontrolled conditions, such as inclement weather, a few sites were less visited. Others were visited in previous years, and we included those observations in our results.

**TABLE 1:** The 38 municipalities surveyed in inland São Paulo state, providing their location regarding the Cerrado, their area, and the number of times and dates each one was visited.

Municipality	Cerrado	Area	Visits	Survey
Iperó	East	5528.25	3	24–26 Apr 2016
Jundiaí	East	38197.26	3	17–19 Nov 2015
Manduri	East	735.93	4	16 Feb 2015; 21–23 Apr 2016
Piraju	East	104.4	3	14–16 Feb 2015
Timburi	East	2101.14	4	17 Feb, 30 Apr, 1–2 May 2015
Andradina	West	228.33	2	13–14 Sep 2021
Braúna	West	186.84	2	11–12 Sep 2021
Caiuá	West	1131.84	3	15–17 Sep 2015
Dracena	West	39.51	3	12–14 Sep 2015
Gália	West	3855.15	6	29–30 Apr, 1 May 2016; 15–17 Jul 2022
Guaraçaí	West	742.41	3	9–11 Sep 2015
Guaraçaí	West	595.71	3	9–11 Sep 2015
Jaú	West	192.06	3	18–20 Apr 2016
Junqueirópolis	West	37.48	1	8 Sep 2015
Marília	West	180.72	2	2–3 Jan 2022
Nova Independência	West	11.61	2	9–11 Sep 2015
Ouro Verde	West	34.38	4	11–14 Sep 2015
Ouro Verde	West	14.94	4	11–14 Sep 2015
Paulo de Faria	West	506.25	4	14–15 Sep 2021; 3–4 Jan 2022
Pompeia	West	3603.6	2	10–11 Sep 2021
Presidente Epitácio	West	536.04	5	14–21 Sep 2015; 7–8 Dec 2015
Presidente Epitácio	West	415.98	4	14–21 Sep 2015; 7–8 Dec 2015
Presidente Epitácio	West	245.43	3	14–21 Sep 2015; 7–8 Dec 2015
Presidente Epitácio	West	201.78	3	14–21 Sep 2015; 7–8 Dec 2015
Presidente Epitácio	West	46.98	2	14–21 Sep 2015; 7–8 Dec 2015
Presidente Venceslau	West	1.35	4	14–17 Sep 2015
Sabino	West	424.71	2	9–10 Sep 2021
Teodoro Sampaio	West	39324.33	7	20–26 Sep 2015
Teodoro Sampaio	West	1285.92	3	6–7 Nov 2015
Tupi Paulista	West	23.72	1	10 Sep 2015
Valparaiso	West	78.48	2	12–13 Sep 2021
Altinópolis	Within	534.51	3	14–16 Sep 2016
Anhembi	Within	1461.33	4	14–17 Nov 2015
Bauru	Within	287	1	17/jul/22
Bofete	Within	19.17	1	16 Feb 2022
Botucatu	Within	775.44	2	14 Feb 2022
Brotas	Within	372.51	4	30 Dec 2014; 2–4 Mar 2016
Lençóis Paulista	Within	442.35	2	12–13 Apr 2016
Luís Antonio	Within	10816.65	3	27–29 Sep 2016
Matão	Within	2415.6	3	28 Feb; 11 and 18 Mar 2016
Pardinho	Within	45	2	16–17 Apr 2022
Pedregulho	Within	1825.65	6	20–22 Ago 2021; 4–6 Jan 2022
Porto Ferreira	Within	786.78	5	27–28 Dec 2013; 29–30 Nov, 1 Dec 2015
Santa Rita do Passa Quatro	Within	317.25	5	27–28 Dec 2013; 29–30 Nov, 1 Dec 2015
São Simão	Within	1038.69	2	6–7 Jan 2021

### 2.3 Historical Records

Collectors and researchers associated with natural history museums (particularly the Museu de Zoologia da Universidade de São Paulo—MZUSP) provided important information via museum specimens. The records of researchers who visited the region before the 1990s were conducted in several inland municipalities. These data are the foundation upon which the distribution of forest birds was established and were brought together by pioneering ornithologists Willis and Oniki (2003), who ventured into several municipalities we now revisited. We also reviewed the historical (pre‐1990s) literature in an AF bird's data paper (Hasui et al. 2018).

### 2.4 Bird Surveys

We used 10‐species lists, in which the observer includes the 10 first species recorded visually or aurally while slowly walking along pre‐existing transects and forest borders. No species can be repeated within a list, but a given species may be present in subsequent lists. The sampling effort is based on the number of accumulated lists (Herzog, Kessler, and Cahill 2002). Surveys occurred between December 2014 and July 2022, beginning approximately 15 min before sunrise and lasting for 2–3 h. Most selected fragments were visited consecutively for three mornings, with a median of 3.0 (1–7) (Table 1), and overall, 729 lists were produced, with a median of 8.5 (2–62) lists per fragment.

### 2.5 Ornithological Online Platforms

We searched for bird species recorded within the state of São Paulo in Brazil's most used online ornithological platforms: eBird (https://ebird.org/) and Wikiaves (https://www.wikiaves.com.br/). The latter requires documented (photographed or recorded) evidence. The eBird platform provides lists and media (photographs and recordings are optional) showing that citizen scientists are reporting birds from the inland forest fragments, yet virtually none of those records includes endemics or forest species. Thus, potential spatial sampling bias does not account for the absence of records of target (endemic) bird species in those fragments.

### 2.6 Analyses

We compared Atlantic Forest endemic bird communities across different periods (pre‐ and post‐1990s). Endemic species richness was compared using sample‐based rarefaction and extrapolation curves based on the Hill number of the diversity order with its parameter of order *q* = 0, which corresponds to species richness (Chao et al. 2014), while the composition of the endemic species community was assessed through two distinct analyses. First, it was determined whether the current community represents a nested subset of that recorded prior to the 1990s using the Nestedness Metric based on Overlap and Decreasing Fill (NODF) (Almeida‐Neto et al. 2008). Next, a permutational multivariate analysis of variance (PERMANOVA) was conducted to evaluate differences in the composition of endemic species between the two periods. Both analyses were performed using the vegan package (Oksanen 2013). To visualise potential changes in these communities over time, Non‐metric Multidimensional Scaling (NMDS) was employed. We combined historical and recent data on species presence and absence for the same municipalities and used the Bray–Curtis dissimilarity as the distance metric to reflect the pairwise dissimilarities between samples. Finally, to evaluate temporal changes in the communities, we used a beta diversity index in the betapart package (Baselga and Orme 2012), which controls for species non‐detection, to determine whether changes were due to nestedness, species turnover or dissimilarity between periods. Sixteen municipalities located within or west of the Cerrado were included in all analyses given they were sufficiently previously surveyed, harbouring ≥ 50 endemic species prior to the 1990s. Analyses were performed in R (R Core Team 2023). Atlantic Forest endemics followed a recent classification (Vale et al. 2018).

## 3 Results

Regarding historical records, museum and literature records from the pre‐1990s indicate the presence of 771 bird species in the state of São Paulo. Of these, 152 (20%) were AF endemics.

In our recent surveys, we recorded 422 bird species in 38 municipalities within and west of the Cerrado. In these inland municipalities, 366 bird species were observed, of which 42 (11%) were AF endemics. The ornithological online platforms indicated the presence of 512 species, with 427 (83%) recorded on eBird and 457 (89%) on Wikiaves. Endemics accounted for 61 (20%) species.

Until the 1990s, coastal municipalities exhibited 77–134 endemic species. Botucatu and Anhembi were the municipalities with the highest species richness within the Cerrado. The municipalities west of the Cerrado with the greatest endemic species richness were Teodoro Sampaio and Gália (Table 2). There are discrepancies regarding the time span in which naturalists and Willis and Oniki visited these localities prior to the 1990s. Thus, our comparisons strictly focused only on the species they did encounter.

**TABLE 2:** Inland municipalities in São Paulo state, south‐eastern Brazil, where Atlantic Forest endemic bird species have been recorded.

	Municipality	Until the 1990s	Early time span	After the 1990s	Inland
1	Botucatu	54	1900–1987	39	Cerrado
2	Anhembi	51	1950–1975	50	Cerrado
3	Itararé	44	1820–1984	26	East
4	Teodoro Sampaio	33	1941–1989	46	West
5	Gália	32	1975–1998	47	West
6	Itirapuã	31	1987–1997	10	Cerrado
7	São Carlos	26	1895–1996	25	Cerrado
8	Timburi	26	1983–1987	35	East
9	Itapeva	25	1901–1984	47	Cerrado
10	Itapura	24	1901–1985	0	West
11	Florínia	23	1943	0	West
12	Lins	23	1914–1985	5	Cerrado
13	Valparaíso	22	1926–1931	2	West
14	Matão	21	1905–1982	21	Cerrado
15	Pirajuí	21	1905	7	West
16	Avaré	20	1963–1997	10	Cerrado

*Note:* Municipality numbers are cross‐referenced to Figure 1. The columns within the dataset denote the number of species documented prior to, and after the 1990s. Time spans refer to the period in which naturalists and early ornithologists obtained information. Inland refers to the municipality's location regarding the Cerrado.

**FIGURE 2:**
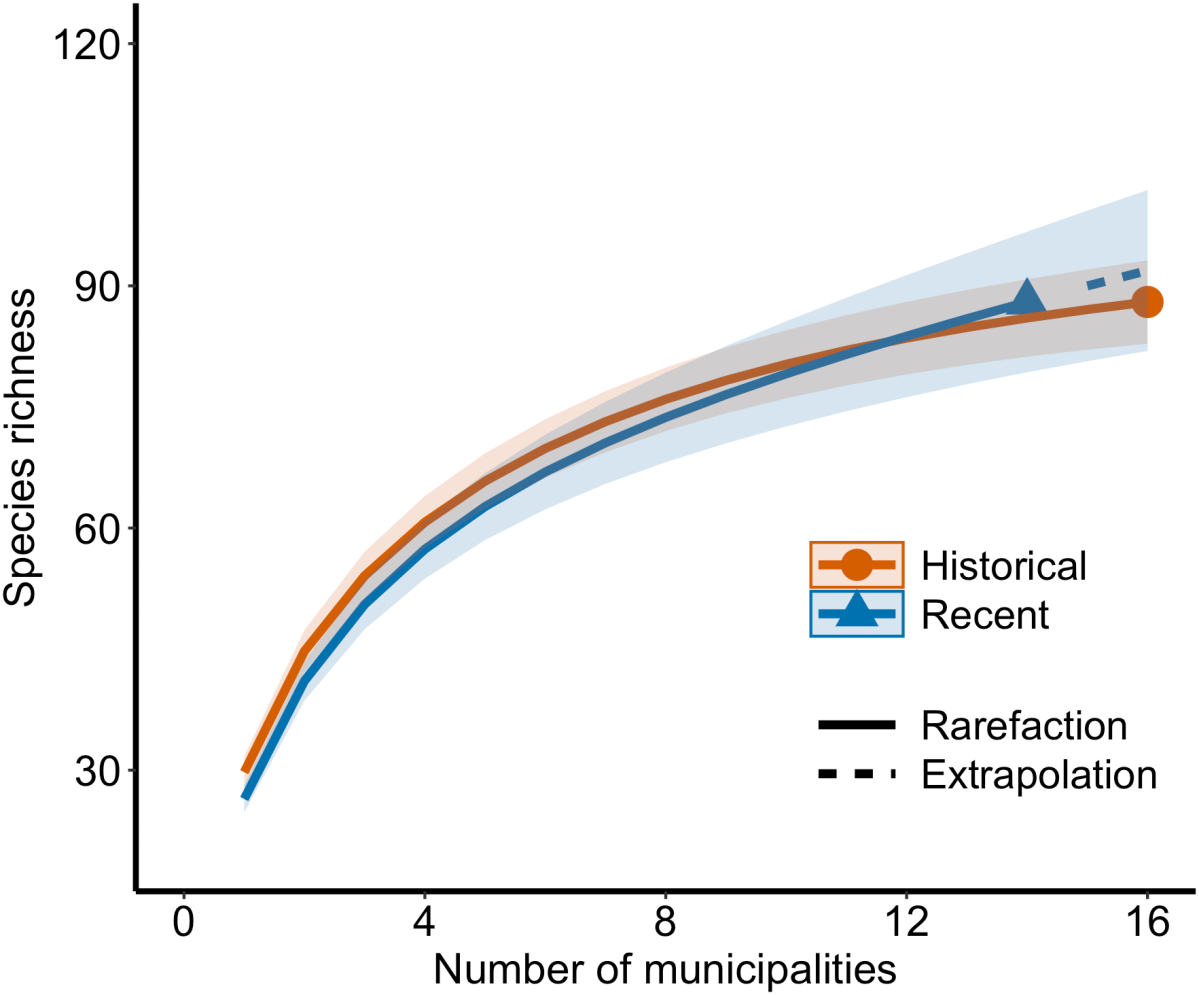
Sample‐based rarefaction and extrapolation curves with Hill numbers of the diversity order from historical (orange) and recent (blue) inventories of Atlantic Forest endemic bird species in inland municipalities in the state of São Paulo, south‐eastern Brazil. The number of recent municipalities with records of endemic species was extrapolated up to 16 municipalities. Shaded areas show 95% confidence intervals.

**FIGURE 3:**
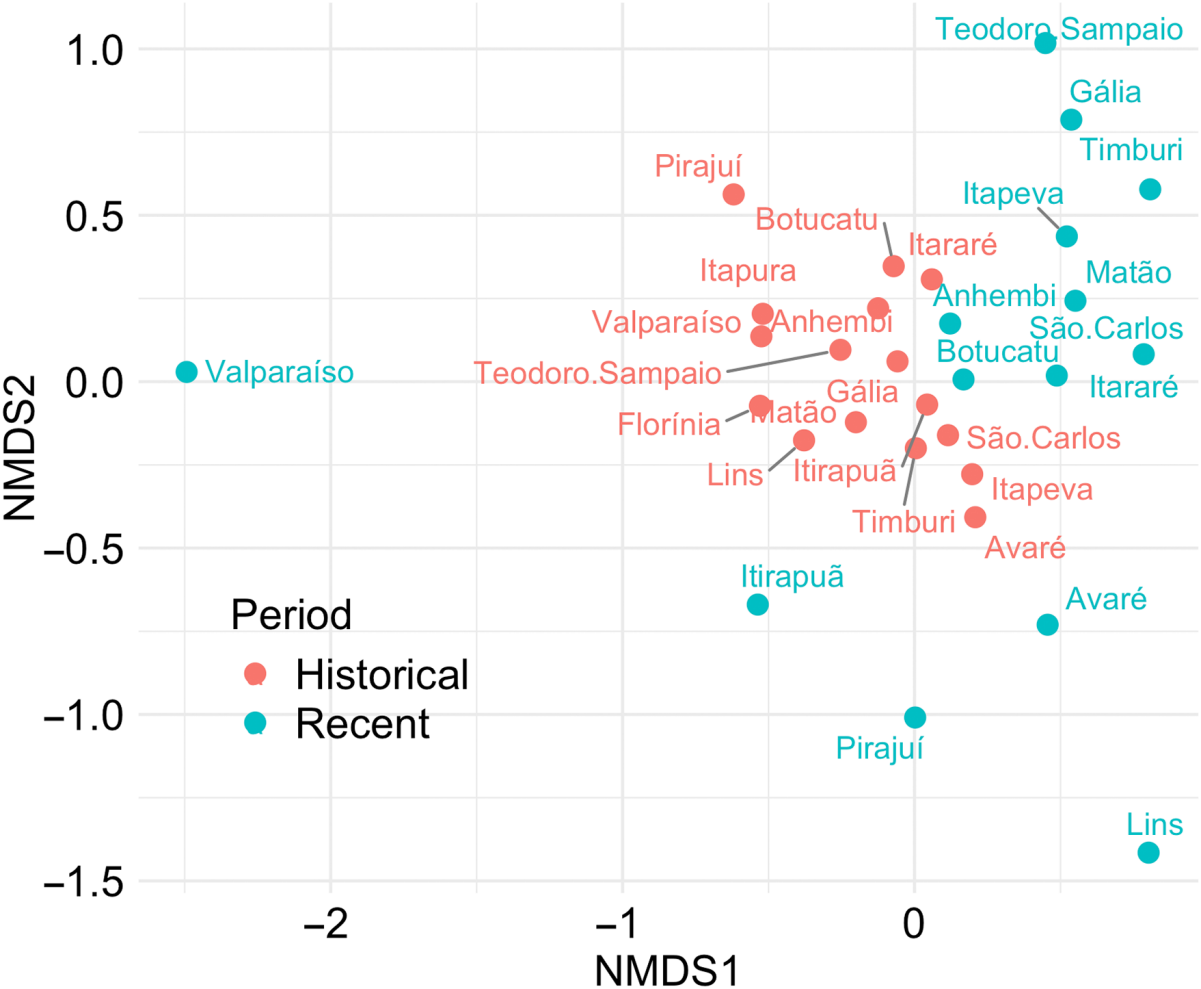
NMDS ordination for Atlantic Forest endemic bird species composition from historical (orange) and recent (blue) surveys in inland municipalities in the state of São Paulo, south‐eastern Brazil.

The sample‐based rarefaction curves demonstrate that the overall endemic species richness is similar between periods. This difference is not significant, given the wide overlap of the 95% confidence intervals between the historical and current rarefaction curves (Figure 2). The NODF analysis indicated that the presence–absence matrix of the period post‐1990s is more nested than the pre‐1990s one (Appendix S1), and that the matrices are more nested than expected by chance (*p* = 0.01). The composition of communities over time differed significantly (*F* = 4.4, *p* = 0.001) according to PERMANOVA. A cluster of the 16 municipalities can be identified prior to the 1990s. For the subsequent period, some municipalities remained close to that cluster, but those with fewer endemic species were less similar and thus distant from the 1990s cluster. For two municipalities, no endemic species were recently found (Table 2, Figure 3). The beta diversity between communities in different periods was 53.7, and the nestedness component indicates that 19.4% of the beta diversity is due to changes in composition between periods. The species turnover component indicates that 28% of the beta diversity is due to species turnover between periods.

**FIGURE 4:**
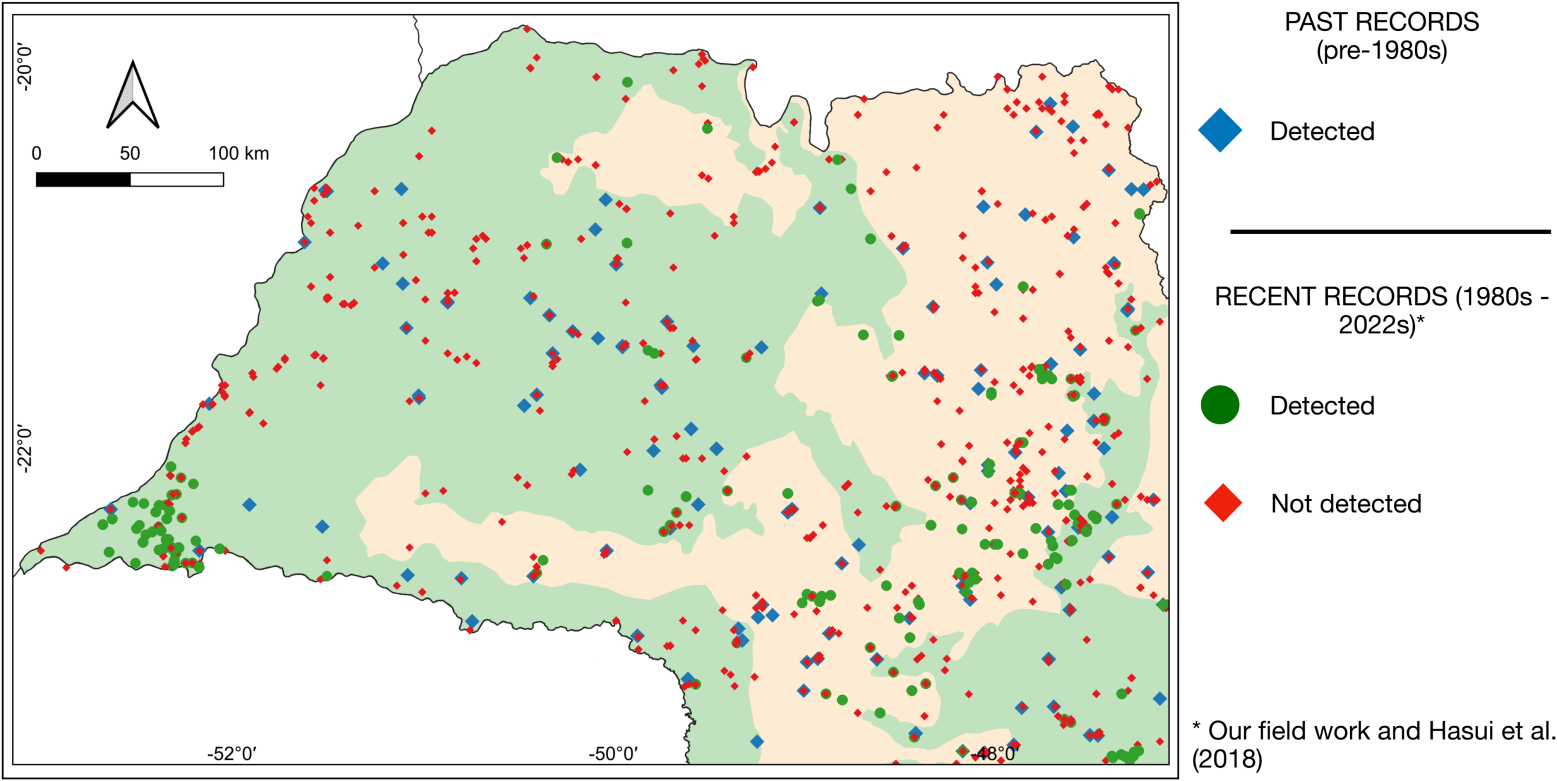
Past and current distribution of 10 Atlantic Forest endemic species. Past records were compiled by Willis and Oniki (2003) and Hasui et al. (2018). Recent records are based on recent surveys and literature review of places where the species were (or were not) detected.

**FIGURE 5:**
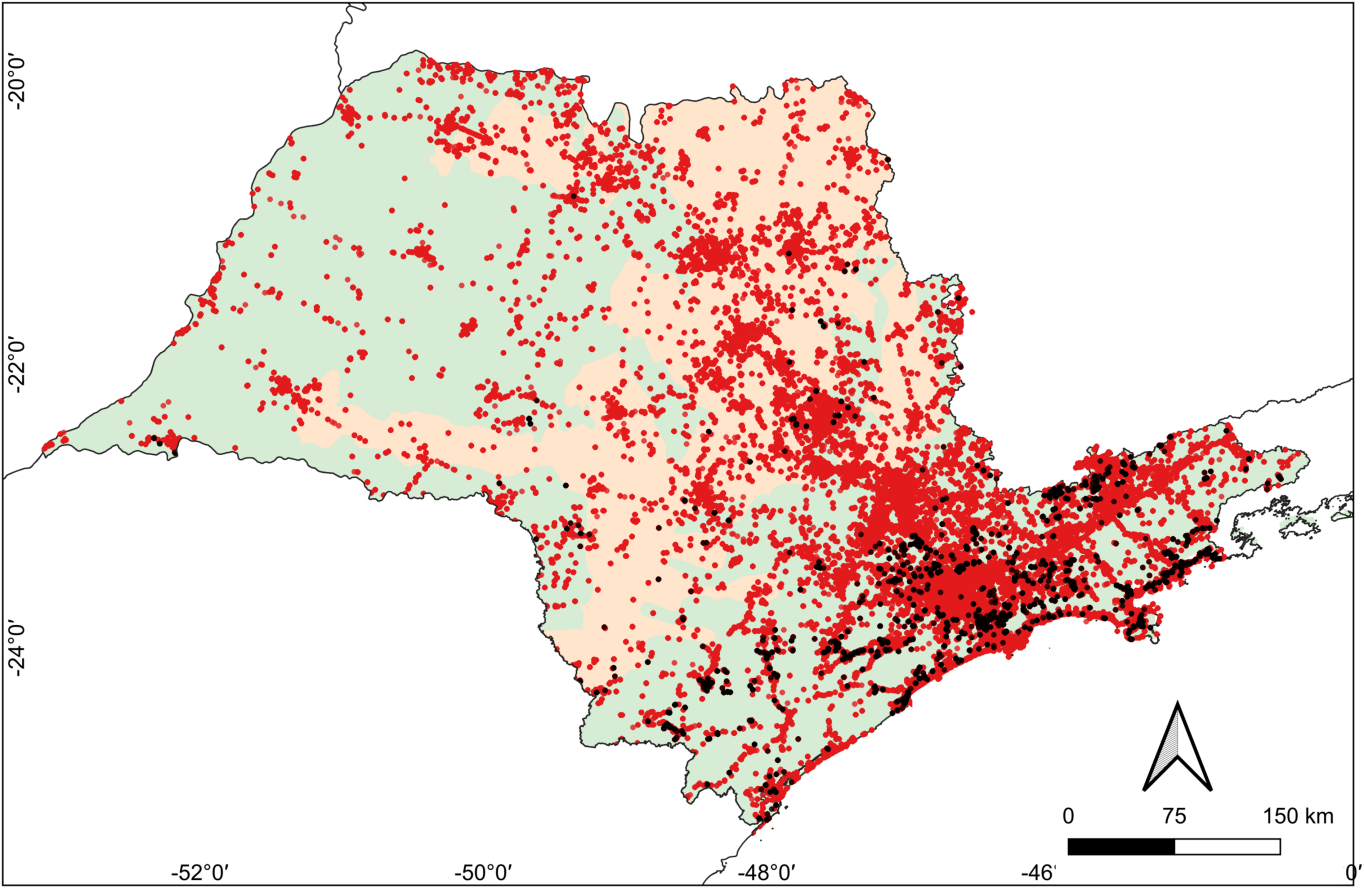
Places where species lists were uploaded in São Paulo on the eBird platform through June 2024 (red), and places where the White‐shouldered Fire‐eye *Pyriglena leucoptera* was recorded (black).

We selected 10 common and abundant AF endemic species from distinct orders, phylogenetic histories and degrees of environmental sensitivity (*Thalurania glaucopis*, *R. dicolorus*, *Pyrrhura frontalis*, *Mackenziaena severa*, *Pyriglena leucoptera*, *Xiphorhynchus fuscus*, *Automolus leucophthalmus*, *Synallaxis ruficapilla*, *Chiroxiphia caudata* and *Tachyphonus coronatus*) whose range included inland São Paulo and which had > 60 historical records prior to the 1990s for a detailed temporal inspection. Historically, these species occurred throughout the state. However, most recent records come from the Cerrado east to the coast, with fewer records west of the Cerrado (Figure 4). The four municipalities that represent recurrent exceptions to this general trend are Teodoro Sampaio (home to Morro do Diabo State Park), Gália (location of the 2180‐ha Caetetus Ecological Station) and Garça (a 320‐ha privately owned reserve 12 km north of Caetetus), and Matão (where a privately owned 2000‐ha reserve exists). Few recent records of endemic species have been noted elsewhere west of the Cerrado. These include another four localities in which a median 5 (1–5) of the 10 mentioned species were detected. This pattern can be clearly illustrated using the White‐shouldered Fire‐eye *P. leucoptera* as an example (Figure 5).

## 4 Discussion

Our compilation of historical data provided an accurate status of AF endemics in semideciduous forests, while our surveys and the review of the literature and ornithological platforms updated their occurrence in São Paulo. Recent studies that inventoried north‐eastern and north‐western AF semideciduous forests often failed to find endemic species (Hasui et al. 2018). Due to the land use practices we previously described, there are essentially no fragments with native vegetation older than 100 years in São Paulo within and west of the Cerrado (Dean 1997; Victor et al. 2005). Thus, these fragments represent the vegetation which have been regenerating over the past 100 years, that is, they now occupy previously clear‐cut places. This left no mature forested habitats, which, in turn, are isolated from each other and from eastern source areas. Some 30 years ago, ornithologists Willis and Oniki (1992) predicted the point of no return for habitat‐specialist species of the fragmented AF. These pioneering researchers carried out bird inventories in many western municipalities from the 1970s to the 1990s. They encountered endemic species that are not currently found (Willis and Oniki 2003) and warned about the implications of losing them (Willis and Oniki 1992).

### 4.1 Non‐Detection of Endemic Species

Museum and pre‐1990s records suggest that recent species richness of endemic species is similar to historical species richness. This result was expected and reflects the fact that the endemics recorded before the 1990s are still detected in notable exceptions of a few large extant forest tracts (Cavarzere et al., in press). They also indicate a generally higher percentage of endemics in coastal rainforests, with numbers decreasing sharply westward. The municipalities of Teodoro Sampaio and Gália did not lose endemic species after the 1990s; in fact, they accumulated more (Table 2). This suggests that endemics are sensitive to fragmentation, may respect a vegetation (humidity) gradient, and that the initial inventories conducted before the 1990s did not detect the entire community. This was also observed for the Atlantic Forest birds from the southern state of Paraná (Januário et al. 2023). Citizen scientists have surveyed nearly all municipalities in São Paulo; thus, spatial sampling bias can be overlooked. Although they tend to favour certain species (Farias, Roper, and Cavarzere 2022), this tendency does not account for inland non‐detections, as target endemic species are frequently reported from eastern forests, even when considering the combination of five independent online ornithological platforms (Forti et al. 2024). Although there are a few records of the 10 endemic species mentioned above (and likely several others) west of the Cerrado on ornithological platforms, their identities should only be confirmed with proper documentation. Many of these records are either accompanied by unclear photographs, allowing only tentative identifications (e.g., WA4080987), or lack documentation.

It has been demonstrated that AF birds, especially endemic species, will eventually disappear due to habitat modification (Ribon, Simon, and Theodoro De Mattos 2003; Silveira, Olmos, and Long 2003). Thus, their detection is not expected in inland São Paulo, given its deforestation background. We hypothesise that forest species were prevented from recolonising the regenerated São Paulo's fragments due to the state's geography. This is based on the available evidence for the southern contiguous state of Paraná. There, semideciduous forests deforestation rates equalled that of São Paulo's, as does the current percentage of forest cover (Gubert Filho 2010; Straube, Willis, and Oniki 2002). In Paraná, there is no Atlantic Forest division across the state, and endemics are still commonly found inland (Januário et al. 2023). These observations suggest that in São Paulo endemics were not able to recolonize inland fragments due to geographic barriers, which are non‐existent in Paraná. In São Paulo, most forest specialists in eastern source areas cannot cross the intervening, equally degraded Cerrado (Barbosa et al. 2017). Also, three major rivers are geographical barriers, at least for some bird families and many understory species (Naka et al. 2022), isolating semideciduous western forests. The Grande River divides all Seasonal Forests of São Paulo and Minas Gerais to the northeast, the Paraná River may impede dispersal to São Paulo from Mato Grosso do Sul to the northwest, and the Paranapanema River hampers movements from Paraná to the east. When considering the three semideciduous forests in São Paulo that still harbour endemic species, it is noteworthy that these areas not only represent the largest remaining forest tracts in their surroundings but also share a common feature: they have never been subjected to clear‐cutting (pers. obs.).

### 4.2 Empty Regenerated Forests

In São Paulo, semideciduous forests west of the Cerrado seem already emptied of endemic bird species. The empty forest paradigm, as conceived, does not apply to them (Redford 1992) because they are not emptied due to overhunting, which leads to the cascading loss of forest species. There, entire communities were extirpated long before they could even be properly studied, and the communities that returned to the newer forest fragments were unlike the original ones. This is consistent with our findings regarding the relevance of nestedness and turnover components, corroborated by other AF human‐altered habitats comparisons (Cavarzere et al. 2022; Vallejos, Padial, and Vitule 2016). Contrasting observations were found for hawkmoths in a preserved Serra do Mar locality (Chiquetto‐Machado, Amorim, and Duarte 2018), from where the composition of endemic birds is known to be quite similar as well (Cavarzere, Moraes, and Silveira 2010). This is particularly problematic given AF ecological patterns and species loss outcomes were mostly based on landscape ecology studies conducted in eastern forests (e.g., Pizo and Tonetti 2020). Small‐scale defaunation is less well‐known and understood but is proving as harmful as well‐established examples of defaunation (Harrison et al. 2013). Even species without conservation issues (Least Concern status) have cryptically declining population trends (Finn, Grattarola, and Pincheira‐Donoso 2023). In São Paulo (and wherever similar patterns hold), State Red Lists of threatened species should consider local and state‐wide population tendencies and consider the well‐preserved eastern AF separately from the fragmented western semideciduous forests that have recovered. For example, the common and abundant White‐shouldered Fire‐eye is not threatened in the Serra do Mar, but it is difficult to find and possibly lost from many or all fragments of semideciduous forests west of the Cerrado in São Paulo. Thamnophilids are a good example here, as AF endemic antbirds do show a high detection probability (Del‐Rio, Rêgo, and Silveira 2015).

Protected areas may no longer resolve the situation. Public nature reserves were established in the late 1970s and early 1980s in then‐existing forest fragments without considering that those fragments had already been modified and regenerated. Thus, forest species were certainly affected, and they remain undetected in recent inventories (Cavarzere, Costa, and Schunck 2023).

### 4.3 The Consequences of Bird Extinctions

Changes in AF endemic bird species have been demonstrated for a well‐studied locality east of the Cerrado and were suggested to have been due to forest modifications over two centuries (Cavarzere et al. 2017). Here, we present evidence of an unprecedented local wave of endemic forest bird extinction in semideciduous AF in São Paulo, over the last 100 years. Many of these species are still abundant in coastal AF, and their commonness may have masked their absence in semideciduous forests west of the Cerrado. As most (80%) of the Brazilian AF is deforested, and most of the remaining fragments are as small and isolated as in São Paulo (Project MapBiomas 2024; Vancine et al. 2024), we strongly suspect the pattern we present here to be widespread. Researchers may have overlooked it due to the paucity of collected specimens and the lack of representative bird inventories before deforestation. It may also reflect the preference for conducting research and monitoring in mature (especially preserved) areas. Thus, even though landscape history strongly influences contemporary AF bird biodiversity (Metzger et al. 2009), numerous smaller and isolated fragments west of the Cerrado remained unexplored, leading to unnoticed extinctions.

Important forested habitats and microhabitats cannot be artificially restored, and large‐scale rewilding through introduction or reintroduction to reconstitute the biota, including birds, is not feasible even in the long term. The best prospects for some of the semideciduous fragments of the Brazilian Atlantic Forest depend on an integrative reforestation programme and reconnection of these fragments, enhancing the chances of recolonisation of plants and animals from the eastern to the western forests.

The next urgent step should be to examine how regenerated forests influence the occupancy and colonisation of endemic species. In addition, this pattern of non‐detections is not restricted to AF endemics. Common, abundant and resilient AF birds such as the Golden‐crowned Warbler *Basileuterus culicivorus* may also go undetected from semideciduous forest fragments where they were formerly common (Hasui et al. 2018; Phelps et al. 2020). Thus, the possibility that the loss of inland species is pervasive in Brazilian semideciduous forests and other terrestrial vertebrates should be properly addressed. Faunal turnover is quite noticeable, as resilient and colonisation‐aggressive species from the Cerrado (e.g., Red‐legged Seriema *Cariama cristata*, Toco Toucan *Ramphastos toco*, Picazuro Pigeon *Patagioenas picazuro*, White‐eyed Parakeet *Psittacara leucophthalmus* and Curl‐crested Jay *Cyanocorax cristatellus*) spread out their distribution by occupying West of the Cerrado, artificially insufflating species richness. Finally, with the collaborative assistance of the citizen science community, the endeavour to identify species commonly found in eastern municipalities could serve as a common purpose for involving ornithologists and bird watchers in advocating for the conservation of AF endemic birds.

## Author Contributions

**Vagner Cavarzere:** conceptualization (lead), data curation (lead), formal analysis (lead), funding acquisition (equal), investigation (lead), methodology (lead), project administration (equal), resources (equal), writing – original draft (lead), writing – review and editing (lead). **Luís Fábio Silveira:** formal analysis (supporting), funding acquisition (equal), investigation (supporting), supervision (equal), writing – original draft (equal).

## Conflicts of Interest

The authors declare no conflicts of interest.

## Supporting information


Table S1.
Figure S1.

## Data Availability

The data that support the findings of this study are available at https://zenodo.org/records/12593831.
