# Supplementary material for: Overlooked Bird Extinctions in Semideciduous Atlantic Forests
Source: Ecol Evol. 2024 Oct 16;14(10):e70388. doi: 10.1002/ece3.70388 (PMC11483593; doi:10.1002/ece3.70388)
Supplement: Supplementary file 1 — Table S1. Figure S1. [file ECE3-14-e70388-s001.docx]

**Overlooked bird extinctions in Semideciduous Atlantic Forests**

**Supplemental material**

Table S1. Number of columns and rows of matrices resulting from a Nestedness Metric based on Overlap and Decreasing Fill (NODF) analysis according to pre- and post-1990s periods.

| Period | Number of columns | Number of rows | NODF |
| --- | --- | --- | --- |
| Pre-1990s | 59.1 | 38.1 | 38.5 |
| Post-1990s | 69.6 | 41.9 | 42.3 |


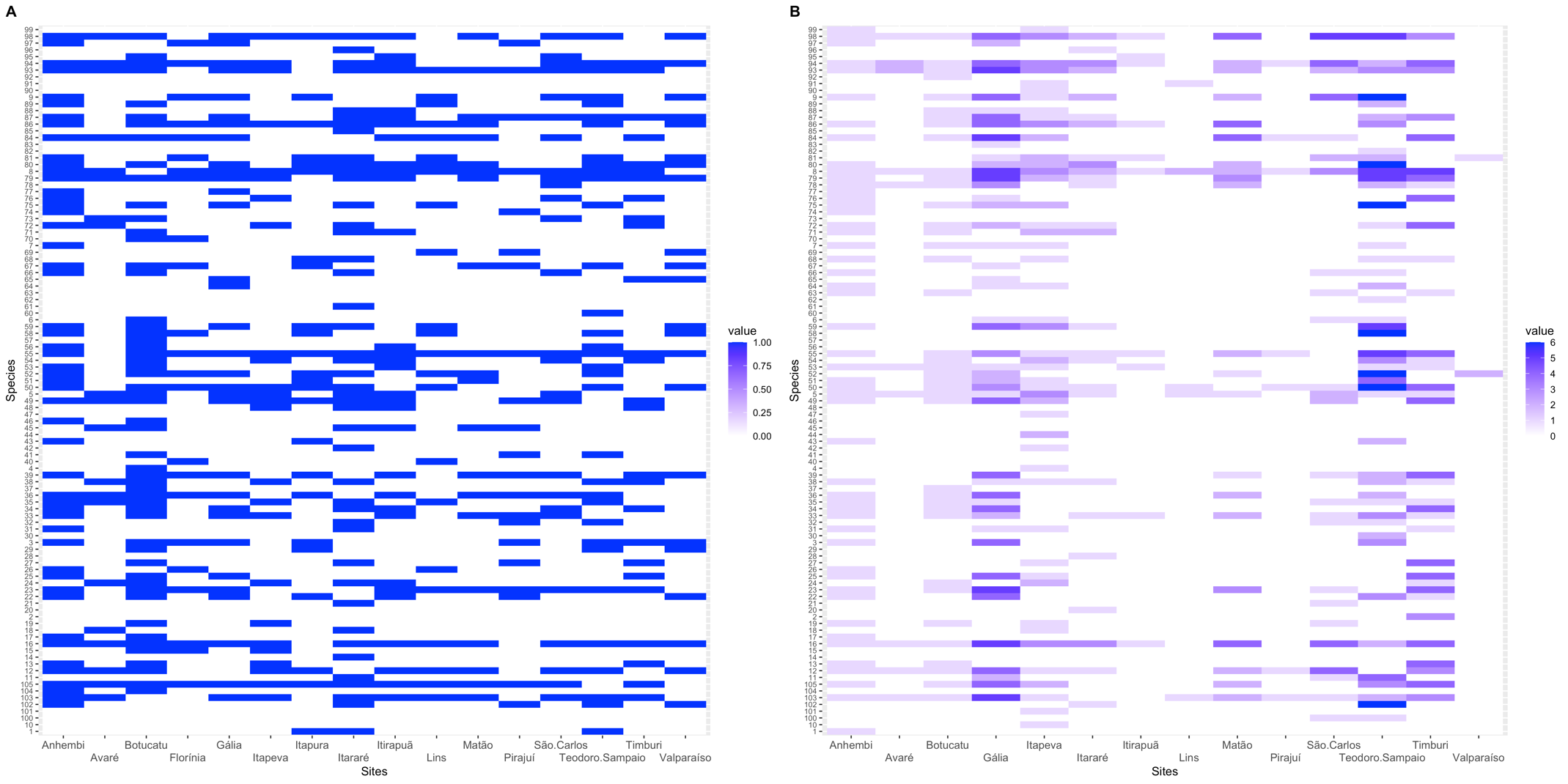


Figure S1. Heatmaps generated by a Nestedness Metric based on Overlap and Decreasing Fill (NODF) analysis for 152 Atlantic Forest endemic bird species according to (A) pre-1990s and (B) post-1990s periods.
